# Use of whole genome sequencing to determine genetic basis of suspected mitochondrial disorders: cohort study

**DOI:** 10.1136/bmj-2021-066288

**Published:** 2021-11-04

**Authors:** Katherine R Schon, Rita Horvath, Wei Wei, Claudia Calabrese, Arianna Tucci, Kristina Ibañez, Thiloka Ratnaike, Robert D S Pitceathly, Enrico Bugiardini, Rosaline Quinlivan, Michael G Hanna, Emma Clement, Emma Ashton, John A Sayer, Paul Brennan, Dragana Josifova, Louise Izatt, Carl Fratter, Victoria Nesbitt, Timothy Barrett, Dominic J McMullen, Audrey Smith, Charulata Deshpande, Sarah F Smithson, Richard Festenstein, Natalie Canham, Mark Caulfield, Henry Houlden, Shamima Rahman(, Patrick F Chinnery, Genomics England Research Consortium, John C Ambrose, Prabhu Arumugam, Roel Bevers, Marta Bleda, Freya Boardman-Pretty, Christopher R Boustred, Helen Brittain, Mark J Caulfield, Georgia C Chan, Greg Elgar, Tom Fowler, Adam Giess, Angela Hamblin, Shirley Henderson, Tim J P Hubbard, Rob Jackson, Louise J Jones, Dalia Kasperaviciute, Melis Kayikci, Athanasios Kousathanas, Lea Lahnstein, Sarah E A Leigh, Ivonne U S Leong, Javier F Lopez, Fiona Maleady-Crowe, Meriel McEntegart, Federico Minneci, Loukas Moutsianas, Michael Mueller, Nirupa Murugaesu, Anna C Need, Peter O’Donovan, Chris A Odhams, Christine Patch, Mariana Buonerimo Pereira, Daniel Perez-Gil, John Pullinger, Tahrima Rahim, Augusto Rendon, Tim Rogers, Kevin Savage, Kushmita Sawant, Richard H Scott, Afshan Siddiq, Alexander Sieghart, Samuel C Smith, Alona Sosinsky, Alexander Stuckey, Mélanie Tanguy, Ana Lisa Taylor Tavares, Ellen R A Thomas, Simon R Thompson, Arianna Tucci, Matthew J Welland, Eleanor Williams, Katarzyna Witkowska, Suzanne M Wood

**Affiliations:** 1 Department of Clinical Neurosciences, School of Clinical Medicine, University of Cambridge, Cambridge, UK; 2 Medical Research Council Mitochondrial Biology Unit, University of Cambridge, Cambridge, UK; 3 East Anglian Medical Genetics Service, Cambridge University Hospitals NHS Foundation Trust, Cambridge, UK; 4 William Harvey Research Institute, Queen Mary University of London, London, UK; 5 Department of Paediatrics, University of Cambridge, Cambridge, UK; 6 Department of Neuromuscular Diseases, UCL Queen Square Institute of Neurology and The National Hospital for Neurology and Neurosurgery, London, UK; 7 Department of Clinical Genetics and Genomic Medicine, Great Ormond Street Hospital for Children NHS Foundation Trust, London, UK; 8 NHS North Thames Genomic Laboratory Hub, Great Ormond Street Hospital for Children NHS Foundation Trust, London, UK; 9 Translational and Clinical Research Institute, Faculty of Medical Sciences, Newcastle University, Newcastle upon Tyne, UK; 10 Northern Genetics Service, Newcastle Hospitals NHS Foundation Trust, International Centre for Life, Newcastle upon Tyne, UK; 11 Department of Clinical Genetics, Guy’s and St Thomas’ NHS Foundation Trust, London, UK; 12 NHS Highly Specialised Services for Rare Mitochondrial Disorders – Oxford Centre, Oxford University Hospitals NHS Foundation Trust, Oxford, UK; 13 Central and South Genome Medicine Service Alliance and Genomics Laboratory Hub, Birmingham Women’s and Children’s NHS Foundation Trust, Birmingham, UK; 14 Institute of Cancer and Genomic Sciences, College of Medical and Dental Sciences, University of Birmingham, Birmingham, UK; 15 Manchester Centre for Genomic Medicine, St Mary’s Hospital, Manchester, UK; 15 Department of Clinical Genetics, University Hospitals Bristol, Bristol, UK; 16 Department of Brain Sciences, London Institute of Medical Sciences, Mansfield Centre for Inovation, Imperial College, Hammersmith Hospital, London, UK; 18 Liverpool Centre for Genomic Medicine, Liverpool Women's Hospital, Liverpool, UK; 19 Genomics England, William Harvey Research Institute, Queen Mary University of London, London, UK; 20 Neurology and Mitochondrial Disorders Genomics Clinical Interpretation Partnership, William Harvey Research Institute, Queen Mary University of London, London, UK; 21 Metabolic Unit, Great Ormond Street Hospital for Children NHS Foundation Trust, London, UK; 22 Mitochondrial Research Group, Department of Genetics and Genomic Medicine, UCL Great Ormond Street Institute of Child Health, London, UK

## Abstract

**Objective:**

To determine whether whole genome sequencing can be used to define the molecular basis of suspected mitochondrial disease.

**Design:**

Cohort study.

**Setting:**

National Health Service, England, including secondary and tertiary care.

**Participants:**

345 patients with suspected mitochondrial disorders recruited to the 100 000 Genomes Project in England between 2015 and 2018.

**Intervention:**

Short read whole genome sequencing was performed. Nuclear variants were prioritised on the basis of gene panels chosen according to phenotypes, ClinVar pathogenic/likely pathogenic variants, and the top 10 prioritised variants from Exomiser. Mitochondrial DNA variants were called using an in-house pipeline and compared with a list of pathogenic variants. Copy number variants and short tandem repeats for 13 neurological disorders were also analysed. American College of Medical Genetics guidelines were followed for classification of variants.

**Main outcome measure:**

Definite or probable genetic diagnosis.

**Results:**

A definite or probable genetic diagnosis was identified in 98/319 (31%) families, with an additional 6 (2%) possible diagnoses. Fourteen of the diagnoses (4% of the 319 families) explained only part of the clinical features. A total of 95 different genes were implicated. Of 104 families given a diagnosis, 39 (38%) had a mitochondrial diagnosis and 65 (63%) had a non-mitochondrial diagnosis.

**Conclusion:**

Whole genome sequencing is a useful diagnostic test in patients with suspected mitochondrial disorders, yielding a diagnosis in a further 31% after exclusion of common causes. Most diagnoses were non-mitochondrial disorders and included developmental disorders with intellectual disability, epileptic encephalopathies, other metabolic disorders, cardiomyopathies, and leukodystrophies. These would have been missed if a targeted approach was taken, and some have specific treatments.

## Introduction

Mitochondrial disorders have emerged as a common cause of inherited metabolic disease, affecting approximately 1 in 5000 people.^1^ They are caused by mutations in genes that primarily affect oxidative phosphorylation and ATP synthesis.^2^ Mitochondria are intracellular organelles that play a pivotal role in cellular energy metabolism. This is achieved by a series of complex enzymes located in the inner mitochondrial membrane that perform oxidative phosphorylation and synthesise ATP. ATP is a chemical source of energy needed for all active cellular processes. The impairment of mitochondrial function tends to affect tissues with high energy demand such as the brain, the peripheral nerves, the eye, the heart, and the peripheral muscles. Clinical diagnosis of mitochondrial disorders is challenging because they can affect a single organ, such as the eye in Leber hereditary optic neuropathy,^3^ or many different systems and can present at any age. Although some patients present with a classic mitochondrial syndrome, such as mitochondrial encephalomyopathy with lactic acidosis and stroke-like episodes, many present with only one or a few of the clinical features (oligosymptomatic cases)^4^ or with an ill defined multisystem disorder.

Mitochondria contain their own DNA in the form of a small 16.5 kb circle of double stranded DNA (mtDNA), which encodes for 13 peptides, two ribosomal RNAs, and 22 transfer RNAs that are essential for synthesising proteins within the organelle. However, the vast majority of proteins making up the mitochondria are encoded by genes in the nucleus and are synthesised in the cytosol before being imported through bespoke import machinery. Mitochondrial disorders can be caused by pathogenic variants in either the mtDNA or the nuclear genome, can follow any inheritance pattern (autosomal dominant, autosomal recessive, X linked, de novo, or maternal), and are highly genetically heterogeneous.^5^ For example, Leigh syndrome, the most common childhood presentation of mitochondrial disease, which usually presents in the first year of life with stepwise loss of skills, is caused by mutations in around 100 nuclear and mtDNA genes.^6^ These challenges often result in a prolonged patient journey from symptom onset to reaching a diagnosis, referred to as a “diagnostic odyssey.”^7^ ^8^ In patients with rare diseases, this typically involves multiple appointments—first in primary care and then with different specialist services—and many investigations, sometimes over many years. One survey found that patients saw an average of eight physicians before having a mitochondrial disease diagnosed, and 70% had a muscle biopsy.^8^

Diagnosis of mitochondrial disease has traditionally relied on an invasive tissue biopsy for biochemical and histochemical analysis,^9^ which can be normal even in patients with a defined genetic diagnosis. Sequencing a pre-defined list of genes known to cause a specific disorder (multi-gene panels) and sequencing of the protein coding regions (exons) of all genes (exome sequencing) have been effective for diagnosing mitochondrial disorders and in discovering new mitochondrial disease genes.^10^ ^11^ ^12^ ^13^ ^14^ ^15^ ^16^ However, making a genetic diagnosis is still not possible in about 40% of cases,^10^ even in highly selected cohorts—hence the need for new approaches.

A definitive genetic diagnosis benefits patients and families,^17^ allowing tailored information about prognosis and treatment, genetic counselling, and access to reproductive options such as prenatal diagnosis (genetic testing during pregnancy, usually by chorionic villus sampling or amniocentesis), pre-implantation genetic diagnosis (the use of assisted reproductive technology and genetic testing of embryos), and mitochondrial transfer (replacing the mother’s mitochondria in an ovum or early embryo with healthy mitochondria from another woman’s donor egg or embryo, used for disorders caused by mtDNA mutations).^18^ Whole genome sequencing is a next generation sequencing technology that is used to sequence the entire genome of an individual. It has the added benefit of being able to diagnose pathogenic mutations affecting the mtDNA and the nuclear genome,^19^ so it has the potential to make a diagnosis in more families and shorten the time to diagnosis.^20^

The objective of this study was to determine whether whole genome sequencing could be used to define the molecular basis of suspected mitochondrial disorders in a national healthcare system in patients assessed in mainstream secondary care as well as tertiary centres. The 100 000 Genomes Project was set up to introduce and embed genomic testing into the mainstream National Health Service (NHS), discover new disease genes, and make genetic diagnosis available for more patients.^21^ Following an initial pilot phase,^22^ patients in the 100 000 Genomes Project were recruited from NHS genomic medicine centres across England. Here, we report the results for 345 patients with a suspected mitochondrial disorder recruited into the main programme between 2015 and 2018.

## Methods

### Participants

Participants with suspected mitochondrial disorders were recruited to the 100 000 Genomes Project (main programme) between 2015 and 2018 with an unexplained multisystem progressive disorder usually involving the central nervous system, the neuromuscular system, or both. All participants provided written informed consent. Eligibility criteria stated that mtDNA and common nuclear genetic causes (eg, *POLG* mutations) should have been excluded (see supplementary methods for full inclusion criteria). All participants recruited under the category “suspected mitochondrial disorder” who had tiering data available in data release v8_2019-11-28 were included in the study. In 2015-18, genetic testing in the UK was arranged through 20 regional genetics laboratories, and there were three NHS highly specialised services for rare mitochondrial disorders. Testing of *POLG* and common mtDNA mutations (m.3243A >G associated with mitochondrial encephalomyopathy with lactic acidosis and stroke-like episodes and maternally inherited diabetes and deafness, m.8344A >G associated with myoclonic epilepsy with ragged red fibres, and m.8993T >G/C associated with Leigh syndrome and neurogenic muscle weakness ataxia and retinitis pigmentosa) in DNA extracted from blood was available through the highly specialised service laboratories. Further testing (such as gene panel testing) was available after discussion with the highly specialised services depending on the patient’s clinical features (supplementary table A).

### Clinical information

The referring clinician provided clinical information against a standardised list of clinical features as yes/no answers (supplementary table B) or by using a standardised vocabulary for describing clinical features encountered in human disease (the Human Phenotype Ontology (HPO)).^23^ HPO classifies clinical features by organ system by using a branching tree incorporating increasing levels of detail. We used modified HPO terms to score participants using the Nijmegen Mitochondrial Disease Criteria^24^ ^25^(supplementary table C), which use clinical features (muscular, central nervous system, and multisystem), magnetic resonance imaging and biochemical features, and muscle biopsy results to give a score out of 12. We classified patients with a total score of 0-1 as being unlikely to have a mitochondrial disorder, those with score 2-4 as a possible mitochondrial disorder, those with score 5-7 as a probable mitochondrial disorder, and those with score 8 or more as definite mitochondrial disorder. One investigator scored all the participants. A second investigator also scored 100 participants independently to develop the modifications for using the Nijmegen Mitochondrial Disease Criteria with HPO terms. For each HPO term, the HPO system was determined by following the branching tree back to the subtype of “phenotypic abnormality.”

### Whole genome sequencing

**Fig 1:**
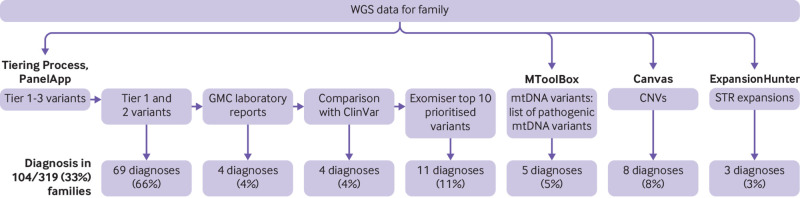
Overview of analyses and sources of diagnoses. Variants in nuclear genes were analysed using Genomics England tiering system. All tier 1 and tier 2 variants were reviewed, and these provided 66% of diagnoses. Another 20% of diagnoses were based on feedback from Genomic Medicine Centre (GMC) laboratories, comparison with Clinvar pathogenic and likely pathogenic variants, and review of top 10 Exomiser prioritised variants. Mitochondrial DNA (mtDNA) variants were analysed separately using in-house pipeline and comparison against list of 89 pathogenic variants, yielding another 5 (5%) diagnoses. Copy number variants (CNVs) accounted for 8% of diagnoses and short tandem repeat (STR) expansions for 3%. WGS=whole genome sequencing

We extracted DNA from peripheral blood and quantified and sequenced it according to a national specification (Illumina TruSeq, HiSeq 2500, and HiSeq X).^26^ ^27^ We aligned reads to the Genome Reference Consortium human genome build 37 (GRCh37) for the earlier participants recruited and GRCh38 for later participants by using Isaac Genome alignment software. Family based variant calling of single nucleotide variants and insertion-deletions for chromosomes 1-22 and X and the mtDNA used the Platypus variant caller,^28^ allowing joint variant calling for all family members and considering the sequence alignments from all family members together. Figure 1 shows the analysis workflow.

### Nuclear variant analysis—single nucleotide variants and small insertion-deletion variants

We analysed genomes in families and classified variants into four “tier” groups according to the probability of the variant being causative.^21^ Tier 1 included loss of function variants (nonsense variants, essential splice donor variants, and essential splice acceptor variants) and de novo missense or splice region variants in genes on the panels applied. Tier 2 included missense and splice region variants in genes on the panels applied. Tier 3 included other rare variants, and a final group of unclassified variants (tier 4) had higher population frequency or the segregation pattern in the family was not consistent with the family history. We chose virtual gene panels according to each participant’s phenotypes, using curated “PanelApp” gene lists, which include causative genes for each disorder generated through crowdsourcing.^29^ This allowed the prioritisation of variants likely to be causative and minimised the reporting of abnormal genetic results that are unrelated to the reason for testing—for example, cancer predisposition genes (referred to as incidental findings). All participants had the mitochondrial panel applied and further panels depended on the phenotypes. Tier 1-3 variants were accessed from the Main Programme v8_2019-11-28. All tiered variants had passed in-house Genomics England quality control.^30^ We also prioritised variants by using Exomiser,^31^ an application that prioritises variants in exome or genome data by using protein interaction networks, clinical relevance, and cross species phenotype comparisons, as well as computational filters for variant frequency, predicted pathogenicity, and pedigree information. We extracted variants classified as pathogenic, likely pathogenic, or pathogenic/likely pathogenic from Clinvar^32^ (3.3.2020) for GRCh38 and GRCh37 and used bedtools intersect (https://bedtools.readthedocs.io) to compare them against tier 1-3 variants to identify previously reported pathogenic and likely pathogenic variants.

### Gene panels applied

We applied the mitochondrial disorders panel in all participants. Other panels applied included undiagnosed metabolic disorders (148 participants), intellectual disability (139), congenital myopathy (77), and hereditary ataxia (60) (see supplementary table D). The mean number of panels applied per participant was 4.7 (range 1-18). A total of 93 different panels were applied to between one and 345 participants.

### Nuclear variant analysis—copy number variants

We detected copy number variant calls by using Canvas software,^33^ on the basis of sequence coverage and both nucleotide and insertion-deletion variant calling. We included only copy number variant calls that had PASS filter status assigned by Canvas and overlapped any gene in PanelApp. We used gencode v29 to annotate copy number variant calls. We evaluated copy number variants that interrupted exons of a PanelApp gene. We manually confirmed all the copy number variants reported as a diagnosis on Integrative Genomics Viewer.

### Nuclear variant analysis—short tandem repeat expansions

We used the ExpansionHunter version 3.2.2 software package for short tandem repeat expansion genotyping.^34^ We assessed 13 loci (*HTT*, *AR*, *ATN1*, *ATXN1*, *ATXN2*, *ATXN3*, *ATXN7*, *CACNA1A*, ​*TBP*, ​*C9orf72*, *FXN, FMR1*, and *DMPK*) by using the coordinates listed in supplementary table E. We visually inspected potentially causative repeat expansions above an established threshold for each locus (supplementary table F) by using pileup plots and re-classified them on the basis of the quality of the reads.^34^

### mtDNA variant analysis

We used an in-house pipeline to call mtDNA single nucleotide variants above an established detection threshold of 1% variant allele frequency (or percentage heteroplasmy level),^35^ after excluding likely errors.^27^ We compared these with a manually curated list of pathogenic mutations with functional evidence supporting pathogenicity.^36^ Our pipeline does not detect large scale mtDNA rearrangements, which usually require targeted mtDNA analysis in DNA extracted from skeletal muscle.

### Clinical review—single nucleotide variants and small insertion-deletion variants

A clinical geneticist reviewed tier 1 and tier 2 variants, the top 10 prioritised variants by Exomiser, ClinVar pathogenic/likely pathogenic variants, and the mtDNA variants (tier 1-3 and from the in-house pipeline) and classified them using internationally accepted criteria for pathogenic variants, likely pathogenic variants, variants of uncertain clinical significance, and likely benign or benign variants (American College of Medical Genetics criteria^37^), incorporating information from gnomAD,^38^ ensembl,^39^ VarSome,^40^ OMIM, and a review of the literature (see supplementary methods for a description of the online resources used). Variant quality and variant allele frequency were checked using Integrative Genomics Viewer.^41^

### Clinical review—copy number variants

We reviewed copy number variants that overlapped at least one exon in a PanelApp gene in DECIPHER. We reviewed information about the type of variant (copy number loss, copy number gain, or loss of heterozygosity), the size of the variant, and the gene content (in particular, haploinsufficient genes and OMIM morbid genes). We also made a comparison with previously observed copy number variants in the general population or in affected individuals.

### Clinical review—short tandem repeat expansions

We reviewed participants with repeat expansions to see whether these were causative, considering the size of the expansion (whether it was a “pre-mutation” or “full mutation”) and the participant’s age and phenotypic features.

### Feedback from Genomic Medicine Centre laboratories and clinical teams

The Genomic Medicine Centre laboratories were commissioned to analyse the tier 1 and tier 2 variants. They filled out an “exit questionnaire” for each family through an online questionnaire. For each family, they fed back whether genetic cause had been identified, with options of “solved,” “partial,” “uncertain,” or “no,” and whether the result was already known through clinical testing. For variants that the clinical scientists had identified as potentially causative, they fed back their variant classification according to the American College of Medical Genetics criteria and whether or not the variant had been confirmed by Sanger sequencing and fed back to the clinician. Feedback from the Genomic Medicine Centre laboratories was available in 277/319 (87%) families (data release v12_21_05_06). We successfully contacted clinicians for further information in 55 (17%) families.

### Overall clinical assessment

We described the molecular diagnosis as “definite,” “probable,” or “possible” on the basis of our overall clinical assessment of whether the variant(s) explained the clinical features, taking into account the American College of Medical Genetics classification of the variant(s), the inheritance pattern, and the clinical fit between the patient’s HPO terms and the reported clinical phenotypes for the gene or variant. For example, we used “probable” for compound heterozygotes for which the phenotype fitted with the disorder, but we classified only one variant as pathogenic/likely pathogenic and the other was a variant of uncertain clinical significance. We described the contribution as “full” or “partial” depending on whether the whole phenotype or only one aspect could be explained by the variant(s). We classified nuclear genes as “mitochondrial” on the basis of the curated PanelApp list,^29^ plus recently discovered genes known to have a direct effect on oxidative phosphorylation.

### Statistical analyses

Statistical analyses were performed in R. We used Fisher’s exact test to compare the number of participants with a genetic diagnosis between children and adults and between singletons and trios/quads. We used Student’s *t* test to compare the mean number of HPO terms in individuals with and without a diagnosis and the number of HPO systems affected. We used Fisher’s exact test to compare the proportion of families with each inheritance pattern between nuclear-mitochondrial diagnoses and non-mitochondrial disorders. We compared the proportion of participants with each HPO system affected between participants with definite mitochondrial diagnoses and definite non-mitochondrial diagnoses, using Fisher’s exact test. We compared the mean Mitochondrial Diagnostic Criteria score between participants with mitochondrial diagnoses, non-mitochondrial diagnoses, and no diagnosis by using a one way analysis of variance and post-hoc Tukey testing.

### Patient and public involvement

The 100 000 Genomes Project has a Participant Panel made up of participants and parents or carers of people involved in the project, which was established in 2016. The panel meets with senior staff from Genomics England and NHS England four times a year. They are asked about project design and help to ensure that participants’ health data are looked after with respect and used in the best interests of the participants. Panel members sit on other committees including the Access Review Committee and the Ethics Advisory Committee. Patients and carers were involved in developing the consent literature and the infographics and information for the public.

## Results

### Demographics

**Fig 2:**
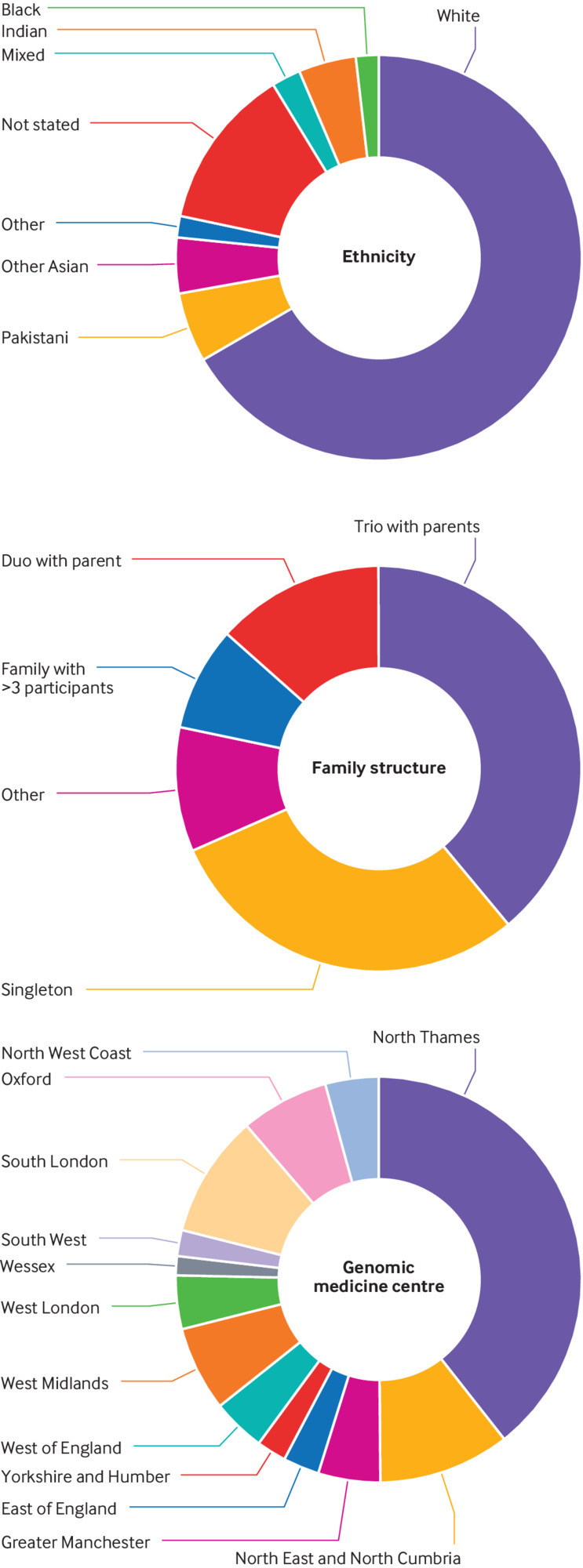
Demographics for participants recruited. Top: ethnicities recorded in participants reflected ethnicity of overall population in England. Middle: most commonly recruited family structures were trios with both parents and singletons. Bottom: participants were recruited from Genomic Medicine Centres across England

Three hundred and forty five affected individuals (186 females, 159 males) were referred with a suspected mitochondrial disorder after exclusion of common causes. They were from 319 families of different reported ethnicities across England (fig 2). Genomic data were available for more than one affected member in 25 families (15 sibling pairs, eight mother and child pairs, one father and child pair, and one mother with two affected children). The median age at enrolment for probands was 25 (interquartile range 10-54; range 0-92) years; 143 (41%) were aged ≤18 at enrolment. No participants withdrew or were lost to follow-up.

### Phenotype data

**Fig 3:**
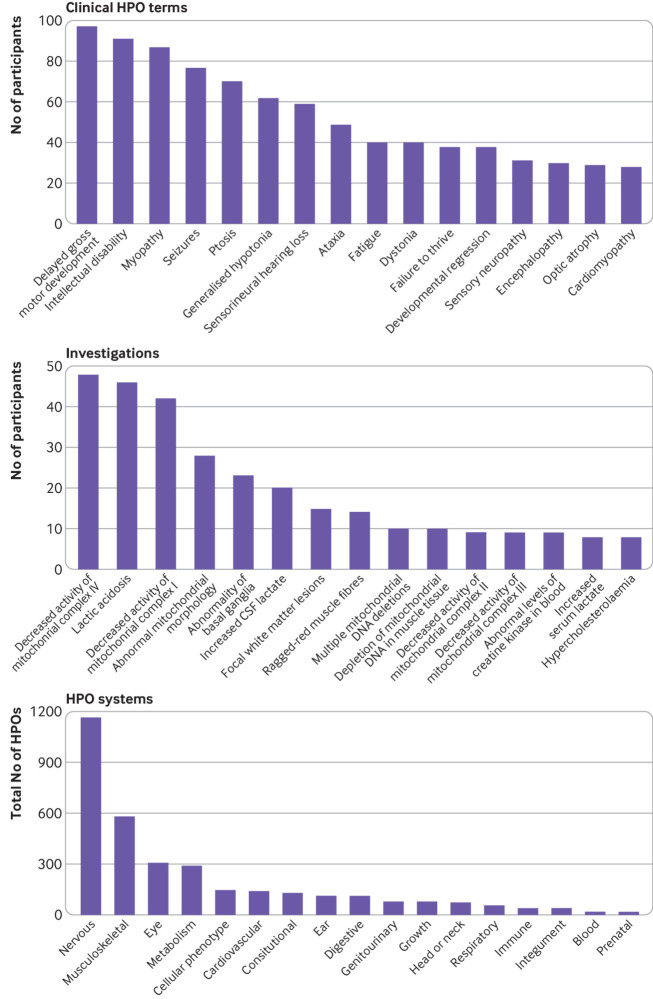
Human Phenotype Ontology (HPO) terms for participants recruited. Top: most commonly recorded clinical HPO terms, including delayed gross motor development, intellectual disability, and myopathy. Middle: most commonly recorded investigation results HPO terms, including decreased activity of mitochondrial complex IV, lactic acidosis, and decreased activity of mitochondrial complex I. Bottom: total number of HPO terms recorded for 345 participants according to ancestor HPO system (some HPO terms have more than one ancestor HPO system)

Phenotypic data were available for 341 participants (missing in four participants). A total of 3095 HPO terms were recorded, with a median of 7 (range 1-39) HPO terms per participant; 806 different HPO terms were used. Figure 3 shows the most common clinical terms, investigation result terms, and HPO systems affected. A median of 4 (range 1-13) HPO systems were affected per participant. Application of the HPO modified Nijmegen score gave a mean total score of 4.30 (range 0-10), with 24/345 (7%) participants classified as being unlikely to have mitochondrial disease (score 0 or 1), 193/345 (56%) as Nijmegen possible (score 2-4), 95/345 (28%) as Nijmegen probable (score 5-7), and 33/345 (10%) as Nijmegen definite (score of ≥8).

### Diagnostic yield

**Fig 4:**
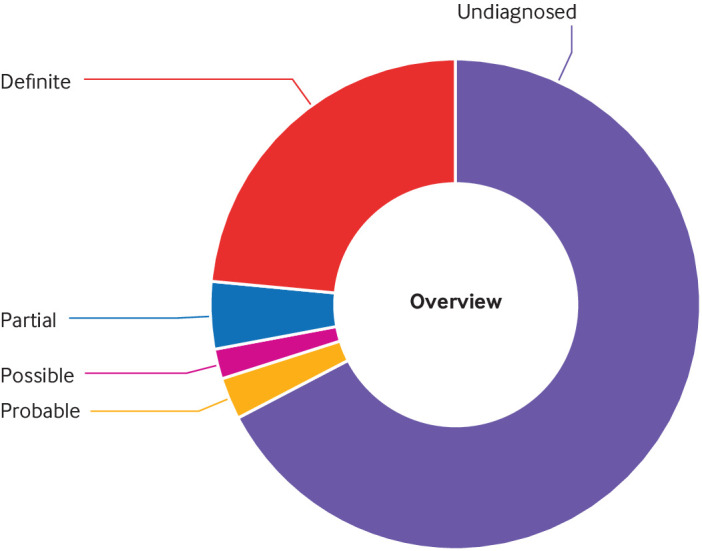
Overview of proportion of families with and without diagnosis

We identified the definite or probable genetic diagnosis in 98/319 (31%) families, with a possible diagnosis in an additional six (2%) (table 1; table 2; fig 1; fig 4). A definite genetic diagnosis was reached in 28% (89/319), including 14 (4%) genetic diagnosis that provided only a partial explanation for their clinical features. Nine (3%) families had a probable diagnosis, and six (2%) had a possible diagnosis. One participant was diagnosed as having two disorders that together fully explained the combination of phenotypes. Most diagnoses (69/104; 66%) came from single nucleotide variants or small insertion-deletion variants on the panels applied (tier 1 or tier 2 variants). An additional 19/104 (18%) diagnoses were made through other analyses of the single nucleotide variants and small insertion-deletion variants (from the Genomic Medicine Centre laboratory reports, comparison with ClinVar, and Exomiser). The copy number variant analysis added eight (8%) diagnoses, and the short tandem repeat analysis added three (3%) diagnoses. Five (5%) diagnoses were made through the mtDNA analysis. Further details of the variant analysis are shown in supplementary table G.

**Table 1:** Variants identified in patients with definite, probable, and possible mitochondrial diagnoses

Family	Age, years	Sex	Contribution	Gene	Variants		ACMG	Inheritance
**Definite**								
1	10	F	Full	*AARS2*		NM_020745.4:c.302G >A p.(Arg101His); homozygous	LP	Biallelic
2	1	M	Full	*AIFM1*		NM_004208.4: c.603_605del p.(Arg201del)	P	XLR; de novo
3	0	F	Full	*ATAD3*		Duplication in ATAD3 gene cluster	P	Monoallelic; de novo
4	0	M	Full	*ATAD3*		Duplication in ATAD3 gene cluster	P	Monoallelic; de novo
5	13	M	Full	*C12orf65*		NM_152269.5:c.210del p.(Gly72fs)	P	Biallelic
						NM_152269.5:c.258_270dupCATCCCCTCAGGC p.(Ile91fs)*	P	
6	6	M	Full	*EARS2*		NM_001083614.2:c.184A >T p.(Ile62Phe);* homozygous	LP	Biallelic
7	2	F	Full	*FBXL4*		NM_001278716.2:c.1641_1642delTG p.(Cys547Ter)	P	Biallelic
						NM_001278716.2:c.141delC p.(Asn48fs) *	P	
8	18	F	Full	*HIBCH*		NM_014362.4:c.1126T >G p.(Phe376Val)*; homozygous	LP	Biallelic
9	22	F	Full	*KARS1*		NM_001130089.1:c.683C >T p.(Pro228Leu)	P	Biallelic
						NM_001130089.1:c.774A >T p.(Arg258Ser)	LP	
10	1	F	Full	*MRPL44*		NM_022915.4:c.467T >G p.(Leu156Arg); homozygous	P	Biallelic
11	23	M	Full	*MRPS25* †		NM_022497.5:c.215C >T p.(Pro72Leu); homozygous	LP	Biallelic
12	42	F	Full	*MT-ATP6*		m.8618dupT; 14% heteroplasmy	P	mtDNA; very low level in mother
13	24	M	Full	*MT-ATP6*		m.8969G >A; 84% heteroplasmy	P	mtDNA; de novo
14	26	F	Full	*MT-ND3*		m.10158T >C; 23% heteroplasmy	P	mtDNA; de novo
15	67	M	Partial	*MT-ND6*		m.14484T >C; homoplasmic	P	mtDNA; unknown
16	18	M	Full	*MT-TE*		m.14674T >C; homoplasmic	P	mtDNA; maternally inherited
17	18	F	Partial	*MT-RNR1*		m.1555A >G; homoplasmic	P	mtDNA; maternally inherited
18	10	F	Full	*MTO1*		NM_012123.4:c.1232C >T p.(Thr411Ile); homozygous	P	Biallelic
19	13	F	Full	*NDUFAF5*		NM_024120.5:c.480-3T >G*	LP	Biallelic
						NM_024120.5:c.827G >A p.(Arg276Gln)*	LP	
20	1	M	Full	*NDUFAF8* †		NM_001086521.2:c.45_52dup p.(Phe18fs)	P	Biallelic
						NM_001086521.2:c.195+271C >T	LP	
21	7	M	Full	*OPA1*		NM_015560.2:c.2708_2711delTTAG (splice acceptor variant)	P	Biallelic
						NM_015560.2:c.1146A >G p.(Ile382Met)	P	
22	11	F	Full	*PDHA1*		NM_000284.4:c.434G >A p.(Cys145Tyr) *	LP	XLD; de novo
23	17	F	Full	*PDP1*		NM_018444.4:c.571C >T p.(Gln191Ter)* homozygous	LP	Biallelic
	13	F						
24	56	M	Full	*POLG*		NM_002693.3:c.1399G >A p.(Ala467Thr); homozygous	P	Biallelic
25	48	F	Full	*RRM2B*		NM_001172477.1:c.242G >A p.(Asp142Asn)	P	Monoallelic; AD
	71	F						
26	0	M	Full	*SCO2*		NM_001169109.1:c.418G >A p.(Glu140Lys)*	P	Biallelic
						NM_001169109.1:c.625_627delTAC p.(Tyr209del)*	LP	
27	0	F	Full	*SCO2*		NM_001169109.1:c.323A >G p.(Asp108Gly)*	LP	Biallelic
						NM_001169109.1:c.281T >C p.(Leu94Pro)*	LP	
28	74	F	Full	*SLC25A4*		NM_002252.4:c.311A >G p.(Asp104Gly)	P	Monoallelic; unknown
29	20	F	Full	*TTC19*		NM_017775.4:c.184+1G >A*	P	Biallelic
						NM_017775.4:c.275_278delCCGA p.(Ala92fs)*	LP	
30	66	M	Full	*TWNK*		NM_021830.5:c.1374G >T p.(Gln458His)	P	Monoallelic; unknown
**Probable**								
31	61	M	Full	*DNM1L*	NM_012062.5:c.239_241delGAG p.(Gly80del)*		LP	Monoallelic; unknown
32	4	F	Full	*ELAC2*	NM_018127.7:c.2009delG p.(Cys670fs)		P	Biallelic
					NM_018127.7:c.2245C >T p.(His749Tyr)*		VUS	
33	7	F	Full	*GFER*	NM_005262.3:c.199delC p.(Arg67fs)		P	Biallelic
					NM_005262.3:c.259-28C >G*		VUS	
34	19	M	Full	*MTFMT*	NM_139242.4:c.626C >T p.(Ser209Leu)		P	Biallelic
					NM_139242.4:c721+5G >A*		VUS	
35	4	F	Full	*RRM2B*	NM_001172477.1:c.578G >A p.(Arg193His)		LP	Biallelic
					NM_001172477.1:c1253C >A p.(Thr418Asn)*		VUS	
36	3	M	Full	*SDHA*	NM_004168.4:c.290G >C p.(Arg97Thr)*		VUS	Biallelic
					NM_004168.4:c.424A >G p.(Met142Val)*		VUS	
**Possible**								
37	18	M	Full	*LONP1*	NM_004793.4: c.1694A >G p.(Tyr565Cys)		VUS	De novo
38	0	M	Full	*PDHA1*	NM_000284.4: c.759+5G >T		VUS	XL (from unaffected mother)
39	56	M	Full	*TOP3A*	NM_004618.5:c.284C >T p.(Ala95Val)*		VUS	Presumed biallelic (parents not tested)
	54	F			NM_004618.5:c.109C >G p.(Leu37Val)*		VUS	

Genetic diagnoses made in 39 families, including age and sex of participants, contribution to phenotype from this gene (full or partial), gene, variants, American College of Medical Genetics (ACMG) variant classification (P=pathogenic, LP=likely pathogenic, VUS=variant of uncertain clinical significance), and inheritance pattern.

AD=autosomal dominant; F=female; M=male; mtDNA=mitochondrial DNA; XL=X linked; XLD=X linked dominant; XLR=X linked recessive.

* Novel variants

† Previously published families.

**Table 2:** Variants identified in patients with definite, probable, and possible non-mitochondrial diagnoses

Family	Age, years	Sex	Contribution	Gene	Variants	ACMG	Inheritance
**Definite**							
40	0	F	Full	*ACTA2*	NM_001614.4:c.536G >A p.(Arg179His)	P	Monoallelic; de novo
41	42	F	Full	*AMACR*	NM_014324.6:c.857delT p.(Ile286fs)*	P	Biallelic
					NM_014324.6:c.437C >T p.(Pro146Leu)*	LP	
42	57	M	Full	*AMACR*	NM_014324.6:c.154T >C p.(Ser52Pro); homozygous	P	Biallelic
43	71	F	Full	*AMACR*	NM_014324.6:c.154T >C p.(Ser52Pro); homozygous	P	Biallelic
44	9	F	Full	*AMPD2*	NM_001368809.2: c.2228T >C p.(Leu743Pro)*; homozygous	LP	Biallelic
45	29	M	Full	*APP*	NM_000484.4:c.2075C >G p.(Ala692Gly)	P	Monoallelic; AD
	58	F					
46	18	M	Partial	*ASL*	NM_000048.4:c.1153C >T p.(Arg385Cys); homozygous	P	Biallelic
47	15	M	Full	*ASXL3*	NM_030632.3:c.3464c >A p.(Ser1155Ter)	P	Monoallelic; de novo
48	15	M	Full	*ATP1A3*	NM_152296.5:c.2452G >A p.(Glu818Lys)	P	Monoallelic; de novo
49	86	F	Full	*ATP1A3*	NM_152296.5:c.2452G >A p.(Glu818Lys)	P	Monoallelic; unknown
50	5	M	Full	*ATP6V1A*	NM_001690.4:c.845A >T p.(Asn282Ile)*	LP	Monoallelic; de novo
51	1	F	Full	*ATXN7* †	Very large CAG repeat expansion	P	Monoallelic
52	44	F	Full	*BBS1*	NM_024649.5:c.1169T >G p.(Met390Arg); homozygous	P	Biallelic
53	2	M	Full	*BCAP31*	NM_001256447.2:c.565C >T p.(Gly189Ter)*	P	XLR; de novo
54	26	F	Full	*C19orf12*	NM_001256047.1:c.245dupC p.(Ala83fs)*	LP	Monoallelic; unknown
55	12	F	Full	*CACNA1A*	NM_001127221.1:c.4177G >A p.(Val1393Met)	LP	Monoallelic; de novo
56	2	M	Full	*CACNA1E*	NM_001205293.3:c.683T >C p.(Leu228Pro)	LP	Monoallelic; de novo
57	12	M	Full	*CTBP1*	NM_001328.3:c.1024C >T p.(Arg342Trp)	P	Monoallelic; de novo
58	11	M	Full	*DOCK6*	NM_020812.4:c.4106+5G >T	LP	Biallelic
	10	F			NM_020812.4:c.1902_1905delGTTC p.(Phe635fs)	P	
59	12	F	Full	*DSP*	NM_004415.4:c.1799T >C p.(Phe600Ser)*	LP	Monoallelic; de novo
60	8	F	Full	*EXOSC3*	NM_016042.4:c.395A >C p.(Asp132Ala); homozygous	P	Biallelic
61	61	M	Partial	*EYA4*	NM_004100.5:c.1741A >T p.(Lys581Ter)*	LP	Monoallelic; unknown
62	2	M	Full	*FIG4*	NM_014845.6:c.447-2A >G*	P	Biallelic
					NM_014845.6:c.827C >T p.(Ser276Phe)*	LP	
63	6	F	Full	*GCDH*	NM_000159.4:c.1204C >T p.(Arg402Trp)	P	Biallelic
					NM_000159.4:c.1304C >T p.(Thr435Met)*	LP	
64	3	F	Full	*HADHA*	NM_000182.5:c.1528G >C p.(Glu510Gln)	P	Biallelic
					NM_000182.5:c.1664T >G p.(Met555Arg)*	LP	
65	10	F	Full	*HK1*	NM_000188.3:c.1334C >T p.(Ser445Leu)	P	Monoallelic; de novo
	8	F					
66	9	M	Full	*HSD17B4*	NM_000414.4:c.590_597dupGATCACGG p.(Met200fs)*	P	Biallelic
					NM_000414.4:c.743G >A p.(Arg248His)*	LP	
67	71	M	Partial	*HTT* †	~40 CAG repeats	P	Monoallelic
68	7	F	Full	*HTT* †	Very large CAG repeat expansion	P	Monoallelic
69	41	M	Partial	*KCNQ4*	NM_004700.4:c.961G >A p.(Gly321Ser)	P	Monoallelic; unknown
70	6	F	Full	*KCNT1*	NM_020822.3:c.1885A >c p.(Lys629Gln)	LP	Monoallelic de novo
71	46	F	Full	*KIF11*	NM_004523.4:c.78-2A >G*	P	Monoallelic; AD
72	29	M	Partial	*KMT2C*	NM_170606.3:c.11669delA p.(Gln3890fs)*	P	Monoallelic; de novo
73	0	F	Full	*MBD5*	NM_001378120.1 deletion of exon 2	LP	Monoallelic; unknown
74	72	M	Full	*MYH2*	NM_017534.6:c.2116G >A p.(Glu706Lys)	P	Monoallelic; unknown
75	57	M	Partial	*MYH7*	NM_000257.4:c.1357C >T p.(Arg453Cys)	P	Monoallelic; unknown
76	13	M	Full	*NARS1*	NM_004539.4:c.1600C >T p.(Arg534Ter)	P	Monoallelic; de novo
77	19	F	Full	*NPHP1*	Gene deletion;homozygous	P	Biallelic
	17	F					
78	3	M	Full	*NPHP1*	Gene deletion; homozygous	P	Biallelic
79	31	F	Partial	*OPTN*	Gene deletion	P	Monoallelic; unknown
80	1	M	Full	*P4HTM*	NM_177939.3:c.659G >A p.(Trp220Ter)*	P	Biallelic
					NM_177939.3:c.569_579del p.(Gln190fs)*	P	
81	36	F	Full	*PDGFB*	Gene deletion	P	Monoallelic; AD
82	11	F	Partial	*PHKB*	NM_000293.3:c.2109delT p.(Ser704fs)*	P	Biallelic
					NM_000293.3:c.2427+977C >T*	P	
83	3	M	Partial	*PKD2*	NM_000297.4:c.1390C >T p.(Arg464Ter)	P	Monoallelic; AD
84	45	F	Full	*PMM2*	NM_000303.3:c.442G >A p.(Asp148Asn)	P	Biallelic
					NM_000303.3:c.305A >G p.(Tyr102Cys)*	LP	
				*AQP2*	NM_000486.6:c.707_720dupTGCTGAAGGGCCTG p.(Glu241fs)*	LP	Biallelic
					NM_000486.6:c.34G >A p.(Ala12Thr)*	LP	
85	2	F	Full	*POGZ*	NM_05100.4:c.2571-2delA*	P	Monoallelic; de novo
86	54	F	Full	*POLR3A*	NM_007055.4:c.2119C >T p.(Gln707Ter)	P	Biallelic
					NM_007055.4:c.1909+22G >A	P	
87	2	M	Full	*PPP2R5D*	NM_006245.4:c.592G >A p.(Glu198Lys)	P	Monoallelic; de novo
88	7	F	Full	*SAMD9*	NM_001193307.1: c.2053C >T p.(Arg685Ter)	LP	Monoallelic; de novo
89	24	M	Full	*SCN2A*	NM_021007.3:c.4480C >A p.(Gln1494Lys)*	LP	Monoallelic; de novo
90	49	F	Full	*SHOC2*	NM_007373.4:c.519G >A p.(Met173Ile)	LP	Monoallelic; unknown
91	56	M	Partial	*SLC20A2*	NM_001257180.2:c.852delC p.(Ile1285fs)*	LP	Monoallelic; unknown
92	25	M	Full	*SLC52A2*	NM_001363118.2:c.368T >C p.(Leu123Pro)	P	Biallelic
					NM_001363118.2:c.916G >A p.(Gly306Arg)	P	
93	11	F	Full	*SOS1*	NM_005633.3.3:c.1294T >C p.(Trp432Arg)	P	Monoallelic; de novo
94	12	M	Partial	*TAB2*	NM_001292034.3: c.-90+1G >C	LP	Monoallelic; de novo
95	7	F	Full	*TANGO2*	Deletion exons 3-9; homozygous	P	Biallelic
96	58	M	Partial	*TTN*	NM_001267550.2:c.59926+1G >A	P	Monoallelic unknown
97	73	M	Full	*TTR*	NM_000371.3:c.407A >C p.(Tyr136Ser)	P	Monoallelic unknown
98	22	F	Full	*ZBTB20*	NM_001164342.2: c.1916G >A p.(Cys639Tyr)*	LP	Monoallelic unknown
**Probable**							
99	59	F	Full	*CTNNB1*	NM_001904.4:c.2315delA p.(Asn772fs)*	LP	Monoallelic; unknown
100	0	M	Full	*MYBPC3*	NM_000256.3:c.1357_158delCC p.(Pro453fs)	P	Biallelic
					NM_000256.3:c.1576G >C p.(Ala526Pro)*	LP	
101	22	F	Full	*PEX16*	NM_057174.2:c.851A >C p.(Tyr284Ser)*; homozygous	VUS	Biallelic
**Possible**							
102	60	F	Full	*MARS1*	NM_004990.4:c.493_495delGAG p.(Glu165del)*	LP	Monoallelic; unknown
103	52	M	Full	*MYH2*	NM_017534.6:c.2387C >A p.(Ala796Asp)*	VUS	Monoallelic; unknown
104	13	M	Full	*MYO9A*	NM_006901.4:c.6796A >T p.(Asn2266Tyr)*	VUS	Biallelic
					NM_006901.4:c.1574A >T p.(Glu525Val)*	VUS	

Genetic diagnoses made in 65 families, including age and sex of participants, contribution to phenotype from this gene (full or partial), gene, variants, American College of Medical Genetics (ACMG) variant classification (P=pathogenic, LP=likely pathogenic, VUS=variant of uncertain clinical significance), and inheritance pattern.

For family 84, full phenotype is explained by two genetic diagnoses. In family 77, NPHP1 homozygous deletion explains phenotype in sibling with severe phenotype (with childhood onset end stage renal disease) and is heterozygous in other sibling.

AD=autosomal dominant; F=female; M=male; XLR=X linked recessive.

* Novel variants

† Previously published families.

### Factors affecting diagnostic yield

**Fig 5:**
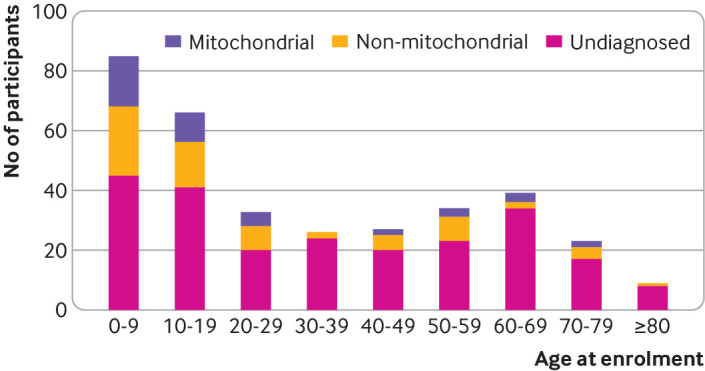
Age distribution of participants at time of enrolment and type of diagnoses made. Diagnostic yield was higher in younger participants, but diagnoses were still being made in patients enrolled in their 70s and 80s

The overall diagnostic rate was 59/186 (32%) in females and 51/159 (32%) in males. Sixty four (45%) of 143 participants recruited aged 18 or under received a diagnosis (any type), compared with 46/202 (23%) participants over the age of 18 (P<0.001). Considering definite diagnoses only, 50/143 (35%) participants under 18 received a definite molecular diagnosis compared with 30/202 (15%) adults over the age of 18 (P<0.001). Figure 5 shows the age profile for diagnosis. Although diagnoses were more frequent in younger people, diagnoses were still being made in participants who were in their 70s or 80s at enrolment.

The diagnostic yield per family (all diagnoses) was 23% (23/102) for singletons, 29% (12/42) for duos with a parent, 42% (62/148) for trios/quads, and 26% (7/27) for other family structures. The diagnostic rate in trios/quads was higher than in singletons (P=0.005). The mean age of singletons was 56 years, compared with a mean age of 12 years for probands in trios/quads.

The mean number of HPOs in participants with any diagnosis (10.0) was slightly higher than in patients with no diagnosis (8.48) (P=0.03). The mean number of HPO systems affected in patients with a diagnosis (4.67) was not significantly different from the mean number of HPO systems affected in those with no diagnosis (4.61) (P=0.82).

### Types of diagnosis

Of the 75 families with a definite diagnosis that fully explains the phenotype, 28 (37%) were in genes known to cause primary mitochondrial disease, including four mtDNA variants and 24 diagnoses in nuclear-mitochondrial genes, whereas 47 (63%) were in non-mitochondrial genes. For families with any genetic diagnosis (including probable or possible diagnoses and partial explanations), 39/104 (38%) were in genes known to cause primary mitochondrial disease, including six mtDNA variants and 30 nuclear-mitochondrial diagnoses, and 65 (63%) were in non-mitochondrial genes. These non-mitochondrial diagnoses included a wide variety of disorders, including developmental disorders with intellectual disability, metabolic disorders, epileptic encephalopathies, Bardet-Biedl syndrome, cardiomyopathies, *MYH2*-related myopathy, and amyloidosis.

### Potentially treatable disorders

Potentially treatable disorders were identified in six participants with a mitochondrial disorder and nine participants with a non-mitochondrial disorder (shown in supplementary table H). However, the evidence base is weak for several of these rare disorders.^42^

### Mitochondrial diagnoses compared with non-mitochondrial diagnoses

#### Inheritance patterns

**Fig 6:**
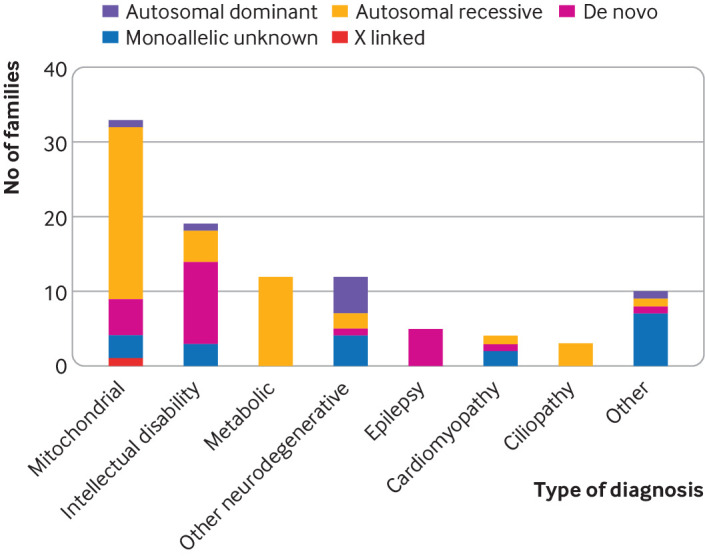
Types of nuclear genetic disorder identified. Inheritance patterns in nuclear mitochondrial disorders and different types of non-mitochondrial disorders. Most families with nuclear mitochondrial disorders showed autosomal recessive inheritance. De novo dominant pathogenic variants were common in families with developmental disorders causing intellectual disability and in epileptic encephalopathies

Seventy per cent (23/33) of families with a nuclear-mitochondrial diagnosis showed an autosomal recessive inheritance pattern, compared with 35% (23/65) of families with non-mitochondrial disorders. Five (15%) families had de novo diagnostic variants in nuclear-mitochondrial genes (*AIFM1*, *LONP1*, and *PDHA1* and two with de novo duplications in the *ATAD3* gene cluster) compared with 29% (19/65) of families with non-mitochondrial disorders having a de novo diagnosis (fig 6), particularly those with intellectual disability or epileptic encephalopathy. The *HK1* variant was de novo in two siblings with presumed germline mosaicism (somatic mosaicism was not detected in either parent). The proportions with each inheritance pattern were significantly different between nuclear-mitochondrial diagnoses and non-mitochondrial disorders (P=0.007).

#### HPO systems affected

**Fig 7:**
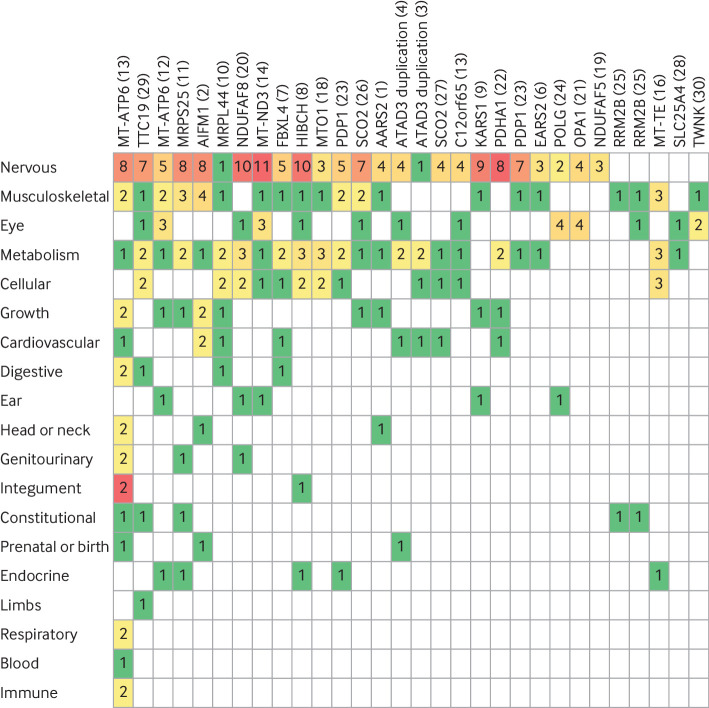
Human Phenotype Ontology (HPO) terms in patients with mitochondrial diagnoses. HPO ancestor systems for HPO terms recorded in participants with definite mitochondrial diagnoses (excluding partial diagnoses). Each column represents one participant (family number in brackets). Each row represents a different HPO ancestor system, listed in order of how frequently they were affected, with nervous system at top. Numbers indicate how many of participant’s HPO terms related to HPO ancestor system (eg, nervous system or musculoskeletal system). Colours go from green through to red as number of terms related to the HPO ancestor system increases

**Fig 8:**
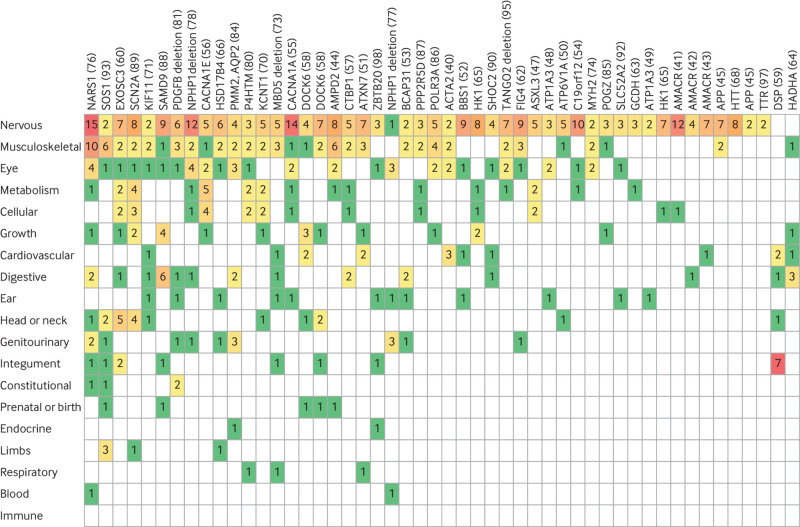
Comparison of Human Phenotype Ontology (HPO) terms in patients with non-mitochondrial diagnoses. HPO ancestor systems for HPO terms recorded in participants with definite non-mitochondrial diagnoses (excluding partial diagnoses). Each column represents one participant (family number in brackets). Each row represents a different HPO ancestor system, listed in order of how frequently they were affected, with nervous system at top. Numbers indicate how many of participant’s HPO terms related to HPO ancestor system (e.g., nervous system or musculoskeletal system). Colours go from green through to red as number of terms related to the HPO ancestor system increases

In our cohort, participants with a mitochondrial diagnosis were more likely to have HPO terms in the metabolism/homoeostasis system (P<0.001), such as increased lactate or decreased mitochondrial complex activities (fig 7), than were those with non-mitochondrial diagnoses (fig 8). Otherwisw, the pattern of HPO systems involved was similar between the two groups.

#### Modified Nijmegen Mitochondrial Disease Criteria scores

**Fig 9:**
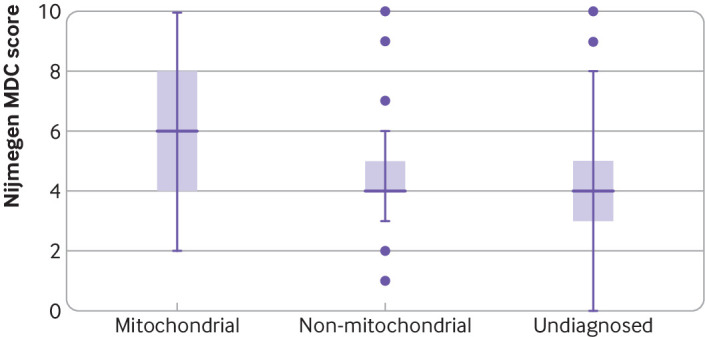
Human Phenotype Ontology modified Nijmegen Mitochondrial Diagnostic Criteria (MDC) scores in participants with confirmed genetic diagnoses of mitochondrial and non-mitochondrial disorders and in participants with no diagnosis. Participants with “probable,” “possible,” and “partial” diagnoses were excluded from this analysis. MDC scores were higher in patients with mitochondrial diagnoses than non-mitochondrial diagnoses or no diagnosis (P<0.05)

Most (22/30; 73%) participants with genetically confirmed mitochondrial diagnoses (nuclear-mitochondrial and mtDNA) had scores in the Nijmegen probable (5-7) and Nijmegen definite (8-12) modified Nijmegen Mitochondrial Disease Criteria category (fig 9), whereas 60% (30/50) of participants with confirmed non-mitochondrial disorders had scores in the possible range (score 2-4). The mean Nijmegen Mitochondrial Disease Criteria score differed between diagnostic groups (P<0.001), including between the mitochondrial diagnoses and the non-mitochondrial diagnoses (P<0.001) and between the mitochondrial diagnoses and patients with no diagnosis (P<0.001) but not between non-mitochondrial diagnoses and patients with no diagnosis (P=0.36). However, 16/50 (32%) participants with confirmed non-mitochondrial disorders had scores in the Nijmegen probable range, and three (6%) had scores in the Nijmegen definite range. The sensitivity and specificity of the Nijmegen Mitochondrial Disease Criteria score in our cohort are shown in supplementary table I. Using Nijmegen definite, the score was highly specific but sensitivity was only 40%, whereas using Nijmegen probable and definite scores, the score was moderately sensitive and moderately specific.

### Muscle biopsy findings and mitochondrial complex activities

One hundred and seventeen (34%) participants had HPO terms relating to muscle biopsy abnormalities, and 73 (21%) had abnormal mitochondrial complex activities. Of patients with definite mitochondrial diagnoses, 15/28 (54%) had muscle biopsy abnormalities, 11/28 (39%) had abnormal respiratory chain complex activities, and two had abnormal pyruvate dehydrogenase complex activity. Of patients with definite non-mitochondrial diagnoses, 14/47 (30%) had muscle biopsy abnormalities and 12/47 (26%) had abnormal respiratory chain complex activities. The patients with non-mitochondrial diagnoses and abnormal respiratory chain complex activities had pathogenic variants in *ASXL3*, *CACNA1E*, *CTBP1*, *EXOSC3*, *HK1*, *KCNT1*, *NPHP1*, *P4HTM*, *PPP2R5D* and *SCN2A*, *SOS1*, and *TANGO2*.

## Discussion

In patients referred for whole genome sequencing with a suspected mitochondrial disorder, a definite or probable diagnosis was identified in 31% (including partial diagnosis in 4%) and a further 2% had a possible diagnosis. The mitochondrial diagnoses were nearly all unique to one family, reflecting the high level of genetic heterogeneity in mitochondrial disorders. Non-mitochondrial disorders were more common than mitochondrial disorders and had features resembling mitochondrial diseases (often referred to as phenocopies). These could be broadly classified as developmental disorders with intellectual disability, metabolic disorders, myopathies, cardiomyopathies, epileptic encephalopathies, leukodystrophies, ciliopathies, amyloidosis, and other neurogenetic disorders, including basal ganglia calcification and neurodegeneration with iron accumulation. Of the non-mitochondrial disorders, 29% were caused by de novo pathogenic variants.

The diagnostic yield was significantly greater in children than in adults. Several possible explanations for this exist. Firstly, children were more likely to be recruited as trios with both parents. This makes analysis more straightforward because de novo variants can be identified, rare familial variants can be filtered out if the parents are clinically unaffected for recessive disorders, and it is possible to check that the variants were inherited from both parents.^43^ Secondly, the most severe phenotypes are seen in children because affected individuals do not survive until adulthood.^44^ Severe phenotypes are the most likely to be caused by single gene disorders. Milder phenotypes overlap with acquired disorders, are less likely to be caused by a single gene defect, and are more common in adults. Thirdly, most adults with genetically proven mitochondrial disorders have mutations in the mtDNA that were excluded by the clinical laboratories before inclusion in this study. Studies of whole exome sequencing in rare disease have also noted a decrease in diagnostic yield with increasing age of the probands.^45^ Despite this, in our study, new genetic diagnoses were made across the whole age spectrum. The oldest patient to receive a genetic diagnosis was recruited at age 86.

The number of people recruited in different age groups was also variable. The highest numbers were recruited in the paediatric age group, with a second peak of patients aged over 60 years. This could be because some adult onset mitochondrial disorders, such as progressive external ophthalmoplegia, take time to develop and become obvious only in later life (progressive external ophthalmoplegia was present in 17/71 (24%) over 60s compared with 16/274 (6%) under 60s). Alternatively, this could reflect the fact that genetic testing had not been offered previously to the older people owing to the financial cost and perceived lack of immediate management implications. Patients in the 30-39 years age group were the least likely to be recruited and had a low diagnostic rate. A common reason for seeking a referral to clinical genetics is for reproductive advice, so we speculate that people in this age group are more likely to have been reviewed by a clinical geneticist and offered up to date genetic testing (such as panel testing) in a clinical setting, meaning that fewer patients did not have a diagnosis and the remaining ones were more difficult to diagnose.

The relatively high frequency of partial diagnoses in this study (4%) may reflect our current knowledge of the phenotypic spectrum of ultra-rare genetic diseases, and some of the features we have not ascribed to the causal variants may actually be due to the underlying mutations. This is likely to be a particular problem for mitochondrial disorders because of their diverse phenotypes, some of which are only just being recognised,^36^ but will become easier as our knowledge base increases.

### Strengths and weaknesses of study in relation to other studies

Our study had several strengths, including the large sample size and the fact that patients were recruited nationally from both secondary and tertiary care, meaning that the findings have a wider relevance than those of studies focused on highly selected groups identified by specialist centres. In addition, the use of HPO terms allowed us to analyse phenotype data in a systematic way, and we contacted clinicians for detailed phenotypic information in selected participants. Our analysis of the nuclear genes included copy number variants and short tandem repeat expansions in addition to single nucleotide variants and small insertion-deletion variants, and we also studied mtDNA variants with a heteroplasmy level >1%. The advantage of using whole genome sequencing rather than whole exome sequencing is that it is easier to pick up copy number variations, repeat expansions, and lower level heteroplasmies. Weaknesses are that we have not explored novel disease genes or variants in the non-coding regions, other than previously published variants, and tracing all family members was not possible.

The previously published study using whole genome sequencing in patients with suspected mitochondrial disease looked at a cohort of 40 children recruited from four paediatric genetic metabolic centres in Australia.^19^ Of these, 34/40 had a probable or definite mitochondrial disorder according to the modified Nijmegen Mitochondrial Disease Criteria score (between 5 and 12) and 28 had abnormal respiratory chain enzyme activities. Analysis of nuclear and mtDNA enabled a definitive genetic diagnosis for 55% of patients, and a likely molecular diagnosis in 67%, with 18% having a non-mitochondrial disorder. The higher diagnostic yield in this study likely reflects the section criteria, which focused on children within trios identified by national specialist clinics,^19^ compared with our more inclusive recruitment criteria whereby around two thirds of our probands were adults and only 46% of families were recruited as trios.

Several studies using whole exome sequencing in patients with suspected mitochondrial disorders have been reviewed recently.^46^ Most of these studies were conducted in highly selected patients seen in specialist centres,^10^ ^12^ ^16^ focusing on childhood onset disorders (apart from Wortmann and colleagues’ study,^14^ which included patients up to 27 years of age). Their stringent recruitment criteria led to higher diagnostic yields (57-68%) and lower numbers of non-mitochondrial diagnoses compared with our study. Previous exome studies of larger cohorts have also tended to have a lower diagnostic yield (35-39%),^14^ ^15^ which is thought to be more reflective of everyday clinical practice.^46^ Non-mitochondrial disorders have been found in some of the previous studies, but in much smaller numbers than in our study. The recruitment criteria in our study were broad, which is reflective of mainstreaming of genomic medicine, and we studied both adults and children. Most participants (64%) did not have muscle biopsy results available at the time of referral, whereas most participants in previous studies had evidence of mitochondrial dysfunction. The more inclusive eligibility criteria have led to us finding a wide range of different genetic diagnoses. The finding that a large number of patients had non-mitochondrial disorders is important because these diagnoses would have been missed if the participants had been investigated only for mitochondrial disorders through muscle biopsy, a specific mitochondrial gene panel, or both. This would have led to missed opportunities for treatment, surveillance, and reproductive management. Our findings highlight the difficulty of diagnosing these rare multisystem disorders clinically and the need to keep an open mind about the differential diagnosis.

Considering other studies of integration of whole genome sequencing into healthcare, Stranneheim and colleagues describe the results for 3219 rare disease patients recruited in Stockholm, Sweden, between 2015 and 2019.^47^ Their study involved much more direct collaboration between academia, healthcare, and their SciLifeLab (which provides whole genome sequencing), which were all located in the same city. This meant that potential diagnoses were discussed and specialist advice was fed back quickly into clinical practice. Specialist clinicians could also access and analyse the data together with clinical scientists. The 100 000 Genomes Project used a different model with the bioinformatics managed centrally and the clinical interpretation of variants done by genomic medicine centre laboratories. Researchers were able to access pseudonymised data. The limited ability of researchers and clinicians to discuss potential diagnoses together and the long turnaround time were disadvantages compared with the Swedish model.

### Unanswered questions and future research

The participants in this study had standard of care NHS genetic testing before enrolment. The use of whole genome sequencing as a first genetic test for suspected mitochondrial disorders has not been directly explored. The only mitochondrial diseases that cannot be diagnosed by whole genome sequencing of DNA extracted from blood are extremely rare muscle specific mtDNA mutations and large mtDNA deletions, which usually have a very specific clinical phenotype. On the basis of known epidemiology,^1^ ^48^ these muscle specific mtDNA mutations and deletions account for approximately 11.5% of patients with a genetically confirmed mitochondrial disorder. Therefore, mitochondrial disorders can be diagnosed by whole genome sequencing of DNA extracted from blood in nearly 90% of patients.^49^ Additionally, whole genome sequencing can diagnose other monogenic disorders that have similar clinical features, so we would expect a very high diagnostic yield. Finally, by carrying out this study we have identified a cohort of patients with suspected mitochondrial disease in whom the likely diagnosis is contained within the whole genome sequencing data but cannot be distinguished from background sequence variation. This will form a useful resource for the discovery of future mitochondrial disease genes that are not in the coding space and thus are not detectable by whole exome sequencing. Large scale mtDNA rearrangements are not reliably detected in DNA extracted from blood in adults, meaning that patients with suspected progressive external ophthalmoplegia and Kearns-Sayre syndrome are investigated by muscle biopsy.^20^ Future research will determine whether deep mtDNA sequencing will detect these large scale mtDNA rearrangements in DNA extracted from blood or another accessible tissue such as urinary epithelium.

### Policy implications

Our findings indicate that whole genome sequencing is a useful diagnostic test in patients with suspected mitochondrial disorders recruited from secondary and tertiary care settings. We recommend that whole genome sequencing should be offered early in the diagnostic pathway in a patient’s local secondary or tertiary care centre and before invasive tests such as a muscle biopsy. Exceptions to this would be patients whose clinical features are highly suggestive of a specific cause that can be confirmed by a single gene test or common mtDNA mutation testing, as well as patients with progressive external ophthalmoplegia, which is diagnosed in most patients by testing mtDNA from a muscle biopsy sample for large scale rearrangements. Referral to a specialised mitochondrial clinic should be considered if whole genome sequencing is uninformative. Further investigations likely to increase the diagnostic yield beyond whole genome sequencing include laboratory studies of mitochondrial function and other “omics” approaches including transcriptomics, which provided an additional diagnosis in 10% of patients with suspected mitochondrial disease in one study,^50^ proteomics, and metabolomics.

The integration of whole genome sequencing into healthcare also has wider policy implications. The relatively high number of patients with probable or possible diagnoses partly reflects the lack of capacity for the functional evaluation of variants of uncertain clinical significance—for example, through splicing assays or Western blotting. Resources should also be made available for the regular reanalysis of whole genome sequencing data, either at specified time intervals in patients without a diagnosis or on a clinician’s request. The reanalysis of exome data has been shown to significantly increase diagnostic yield, mainly owing to newly discovered disease genes.^51^ Finally, rapid trio whole genome sequencing should be offered in acutely unwell individuals with suspected mitochondrial disorders,^17^ so that the results can help to guide clinical management. In the UK, rapid trio exome sequencing is available only for acutely unwell children.

## What is already know on this topic

Mitochondrial disorders are among the most common inherited diseases, but a genetic diagnosis is not possible in around 40% of patients, limiting genetic counselling and prevention
Whole genome sequencing (WGS) has the potential to shorten the “diagnostic odyssey” for patients with suspected mitochondrial disorders
Use of WGS in a national healthcare system has not been previously investigated

## What this study adds

After exclusion of common genetic causes, WGS identified a definite or probable genetic diagnosis in an additional 31% of patients, and another 2% had possible diagnoses
62.5% of the families with a new diagnosis had a non-mitochondrial disorder, showing that a wide genomic approach is more useful than a targeted panel testing approach
The new diagnoses included treatable disorders

## Data Availability

100 000 Genomes Project data are available to researchers and clinicians through joining a Genomics England Clinical Interpretation Partnership) (www.genomicsengland.co.uk/about-gecip/joining-research-community/).

## Web extra

Extra material supplied by authors

Web appendix: Supplementary materials

Web appendix: Supplementary table G

## Notes

Contributors: KRS, RH, and PFC designed the study. PFC and RH supervised the study. WW, CC, AT, and KA did the bioinformatic analysis. KRS, RH, AT, RDSP, RQ, EB, MH, EC, JS, PB, DJ, LI, CN, TB, AS, CD, SFS, RF, HH, SR, and PFC recruited patients. KRS, RH, AT, TR EA, CF, DM, and PFC did clinical interpretation of genetic variants. MC was the chief scientific officer for Genomics England and oversaw the 100 000 Genomes Project. KRS and PFC wrote the manuscript. All authors reviewed and revised the manuscript. PFC is the guarantor. The corresponding author attests that all listed authors meet authorship criteria and that no others meeting the criteria have been omitted.

The lead author (the manuscript’s guarantor) affirms that the manuscript is an honest, accurate, and transparent account of the study being reported; that no important aspects of the study have been omitted; and that any discrepancies from the study as planned (and, if relevant, registered) have been explained.

Dissemination to participants and related patient and public communities: We will distribute the article to clinicians and advocacy groups including the Lily Foundation, Muscular Dystrophy UK, and the United Mitochondrial Disease Foundation. We will distribute findings on social media and a plain language summary on the Cambridge Clinical Mitochondrial Research Group website (https://www-neurosciences.medschl.cam.ac.uk/mitocamb/). We will issue a press release through the University of Cambridge and Genomics England.

Provenance and peer review: Not commissioned; externally peer reviewed.
