# Supplementary material for: Use of whole genome sequencing to determine genetic basis of suspected mitochondrial disorders: cohort study
Source: BMJ. 2021 Nov 4;375:e066288. doi: 10.1136/bmj-2021-066288 (PMC8565085; doi:10.1136/bmj-2021-066288)
Supplement: Supplementary file 1 — Web appendix: Supplementary materials [file schk066288.ww1.pdf]

Supplementary Materials

Supplementary Methods

I. Inclusion Criteria

Mitochondrial Inclusion Criteria

Unexplained multi-system progressive disorder usually involving the central nervous system and/or neuromuscular system. Contributory laboratory findings may include but are not restricted to:

- Characteristic brain MR imaging, e.g. brainstem or basal ganglia involvement, infarction not confined to typical vascular territory, leukoencephalopathy
- Raised serum, CSF or urinary organic acid biomarkers (e.g. lactate, 3-methylglutaconic acid)
- Evidence of mitochondrial dysfunction in diagnostic biopsies including histochemical (COX-deficient fibres, ragged red fibres) and biochemical (respiratory chain deficiencies) markers of disease pathology.

Mitochondrial Exclusion Criteria

- Mitochondrial DNA and common nuclear genetic causes (e.g. *POLG*) excluded (m.3243A>G, POLG)

Prior Genetic Testing Guidance

- Results should have been reviewed for all genetic tests undertaken, including disease-relevant genes in exome sequencing data. The patient is not eligible if they have a molecular diagnosis for their condition.
- Genetic testing should continue according to routine local practice for this phenotype regardless of recruitment to the project; results of these tests must be submitted via the 'Genetic investigations' section of the data capture tool to allow comparison of WGS with current standard testing.

Mitochondrial prior genetic testing genes

Testing of the following genes should be carried out PRIOR TO RECRUITMENT where this is in line with current local practice:

- Mitochondrial DNA

- Common nuclear genetic causes as appropriate

II. Web based resources used for variant interpretation

PanelApp

https://panelapp.genomicsengland.co.uk

PanelApp[1] shows the current virtual gene panels used by Genomics England. The ‘green’ genes / genomic entities have been confidently associated with a monogenic disorder and are used in genome interpretation.

ClinVar

https://www.ncbi.nlm.nih.gov/clinvar/

ClinVar[2] is a publicly accessible archive of human genetic variants with the interpretation of their clinical significance for a disease or phenotype. Tired variants which were classified as pathogenic, likely pathogenic or pathogenic/likely pathogenic were identified by our analysis.

gnomAD

https://gnomad.broadinstitute.org

gnomAD[3] provides summary data from large scale sequencing projects. The v2.1.1 dataset was used for genomes with reads aligned to the Genome Reference Consortium human genome build 37 (GRCh37) and the v3.1.1 dataset for genomes aligned to genome build 39 (GRCh38). gnomAD was used to assess population frequency of variants.

ensembl

http://www.ensembl.org/index.html

The ensembl[4] Variant Effect Predictor was used to aid interpretation of variants.

VarSome

https://varsome.com

VarSome[5] was used to aid interpretation of variants and help identify literature about specific variants.

OMIM

https://www.omim.org

Information from OMIM (Online Mendelian Inheritance in Man) was used in assessing phenotypic match between genes and participants' human phenotype ontology terms.

Integrative Genomics Viewer (IGV)

IGV is a tool for visual exploration of genomic data[6]. Variant quality and variant allele frequency were check using IGV for all potentially causative variants.

DECIPHER

https://www.deciphergenomics.org

DECIPHER is an online database used by the clinical community to share and compare phenotypic and genotypic data. For copy number variants, the DECIPHER Genome Browser shows the genes involved, their haploinsufficiency scores and whether they are disease genes in OMIM. It also shows similar CNVs from DECIPHER participants, variants in ClinVar, defined CNV syndromes (from DECIPHER and GeneReviews) and population data such as gnomAD structural variants.

Supplementary Results

Diagnostic yield using different reference sequences

58 participants were aligned to GRCh37 only, 11 participants had results available separately for GRCh37 and GRCh38 and 276 participants were aligned to GRCh38 only. The diagnostic yield was 24/58 in participants aligned to GRCh37 and 82/276 in participants aligned to GRCh38 ($p = 0.09$), indicating that the reference sequence did not have a significant impact on the number of diagnoses.

Diagnostic yield by ethnicity

The diagnostic yield was 17/50 (34%) in people of Asian ethnicities, 1/6 (17%) in people of black ethnicities, 3/7 (43%) in people of mixed ethnicities, 59/230 (26%) in people of white ethnicities and 2/6 (33%) in people with other ethnicities. There was no significant difference in diagnostic yield between people of different recorded ethnicities ($p=0.51$). There were 46 people who did not have their ethnicity recorded – and the diagnostic yield was 28/46 (61%) in this group. The reason why ethnicity was not recorded in these individuals is not clear.

.

Supplementary Table A – Mitochondrial Genetic Testing in UK National Genomic Test Directory

Genetic testing in the UK has been centralised into Genomic Laboratory Hubs and the tests which can be requested by clinicians have been standardised using the National Genomic Test Directory. The clinical indication, target/genes and test method for testing for suspected mitochondrial disorders are shown below. The top three tests are for use on DNA extracted from a muscle biopsy sample, and the rest are used with DNA extracted from blood.

**Clinical Indication**	**Target/Genes**	**Test Method**
Possible mitochondrial disorder – mitochondrial DNA rearrangement testing	Mitochondrial genome	Other
Possible mitochondrial disorder – whole mitochondrial genome sequencing	Mitochondrial genome	Other
Possible mitochondrial disorder – mitochondrial DNA depletion testing	Mitochondrial genome	Other
POLG-related disorder	Common POLG mutations	Targeted mutation testing
POLG-related disorder	POLG	Single gene sequencing >=10 amplicons
Pyruvate dehydrogenase (PDH) deficiency	Pyruvate dehydrogenase (PDH) deficiency	Medium panel
Mitochondrial liver disease, including transient infantile liver failure	Mitochondrial liver disease	Small panel
MERRF syndrome	Common MERRF mutations	Targeted mutation testing
NARP syndrome or maternally inherited Leigh syndrome	*MT-ATP6, MT-ND6*	Single gene sequencing <10 amplicons
NARP syndrome or maternally inherited Leigh syndrome	m.8993T>C/G	Targeted mutation testing
Mitochondrial DNA maintenance disorder	Mitochondrial DNA maintenance disorder	WES or Medium Panel
Mitochondrial disorder with complex I deficiency	Mitochondrial disorder with complex I deficiency	WES or Medium Panel
Mitochondrial disorder with complex II deficiency	Mitochondrial disorder with complex II deficiency	WES or Small Panel
Mitochondrial disorder with complex III deficiency	Mitochondrial disorder with complex III deficiency	WES or Small Panel
Mitochondrial disorder with complex IV deficiency	Mitochondrial disorder with complex IV deficiency	WES or Small Panel
Mitochondrial disorder with complex V deficiency	Mitochondrial disorder with complex V deficiency	WES or Small Panel

Possible mitochondrial disorder - nuclear genes	Possible mitochondrial disorder - nuclear genes	WES or Large Panel
Mitochondrial neurogastrointestinal encephalopathy	*TYMP*	Single gene sequencing >=10 amplicons
Thiamine metabolism dysfunction syndrome 2	*SLC19A3*	Single gene sequencing >=10 amplicons
Mitochondrial Complex V deficiency, TMEM70 type	*TMEM70*	Single gene sequencing >=10 amplicons
Maternally inherited cardiomyopathy	m.4300A>G	Targeted mutation testing
Leber hereditary optic neuropathy	Three common LHON variants	Targeted mutation testing
Leber hereditary optic neuropathy	Optic neuropathy	Medium panel

Supplementary Table B – Proforma for recording phenotypic information

This proforma was sent to clinicians to document which phenotypes were present in the participants who they recruited. Each phenotype was marked as present, absent or known, and there was free space to add phenotypes which were not covered on the list.

**Phenotype Description**	**Phenotype Present**		
	Unknown	Yes	No
Lactic acidosis			
Increased CSF lactate			
Abnormal mitochondrial morphology			
Depletion of mitochondrial DNA in muscle tissue			
Multiple mitochondrial DNA deletions			
Decreased activity of mitochondrial complex I			
Decreased activity of mitochondrial complex II			
Decreased activity of mitochondrial complex III			
Decreased activity of mitochondrial complex IV			
Decreased activity of the pyruvate dehydrogenase (PDH) complex			
Intellectual disability			
Delayed gross motor development			
Developmental regression			
Failure to thrive			
Progressive external ophthalmoplegia			
Ptosis			
Retinitis pigmentosa			
Optic atrophy			
Sensorineural hearing impairment			
Cardiomyopathy			
Hepatic failure			
Exocrine pancreatic insufficiency			
Proximal tubulopathy			
Generalized hypotonia			
Myopathy			
Ragged-red muscle fibres			
Sensory neuropathy			
Dementia			
Encephalopathy			
Seizures			
Ataxia			
Dystonia			
Abnormality of extrapyramidal motor function			
Stroke-like episodes			
Abnormality of the basal ganglia			

Cont.

**Phenotype Description**	**Phenotype Present**		
	Unknown	Yes	No
Leukodystrophy			
Abnormality of the internal capsule			
Cerebellar atrophy			
Aplasia/Hypoplasia of the cerebellum			
Focal white matter lesions			
Sideroblastic anemia			
Multiple lipomas			

ADDITIONAL PHENOTYPING

**Phenotype Description**	**Phenotype Present**		
	Unknown	Yes	No

Supplementary Table C – Mitochondrial Disease Criteria (modified for use with Human Phenotype Ontology terms)

The Nijmegen criteria[5–7] were modified for use with Human Phenotype Ontology[8,9] terms. The Human Phenotype Ontology terms are arranged in a directed acyclic graph and terms are connected by class-subclass relationships. *denotes that this HPO term or any term inherited from this 'root' term can be used in the score.

Patients with a total of 0-1 were classified as being unlikely to have a mitochondrial disorder, score 2-4 : possible mitochondrial disorder, score 5-7: probable mitochondrial disorder and score 8 or more as definite mitochondrial disorder.

I. Clinical Signs and symptoms 1 point/symptom (max 4)

**Description**	**HPO Terms**
Muscular Presentation (max 2 points)	
Ophthalmoplegia (2 points)	Ophthalmoplegia, External ophthalmoplegia*, Progressive ophthalmoplegia
Facies myopathica	Myopathic facies
Ptosis	Ptosis*
Exercise intolerance	Exercise intolerance, Postexertional malaise, Exercise-induced myalgia, Exercise-induced muscle stiffness, Fatigue, Chronic fatigue
Muscle weakness	Muscle weakness*, Myopathy, Muscular hypotonia*
Rhabdomyolysis	Rhabdomyolysis*
Motor developmental delay	Delayed gross motor development
Abnormal EMG	EMG:myopathic abnormalities
Neurological Presentation (max 2 points)	
Developmental delay or ID	Neurodevelopmental delay*, Intellectual disability (Global developmental delay – counted for developmental delay and for speech delay)
Speech delay	Delayed speech and language development
Loss of skills	Developmental regression

Stroke-like episode	Stroke-like episode
Migraine	Migraine*
Seizures or encephalopathy	Seizures*, Encephalopathy*
Myoclonus	Myoclonus
Cortical blindness	Cerebral visual impairment
Pyramidal signs/ Spasticity	Spasticity*, Hyperreflexia*
Extrapyramidal signs/ Dystonia	Dystonia*, Abnormality of extrapyramidal motor function*
Ataxia	Ataxia*<br>(spastic ataxia – counted for spasticity and for ataxia)
Neuropathy	Peripheral neuropathy*
Multisystem (max 3)	
Haematology/ Immune	Abnormality of blood and blood forming tissues* (excluding neoplasm)<br>Abnormality of immune system*
GI tract	Abnormality of the gastrointestinal tract* (exclude neoplasm)
Endocrine	Abnormality of the endocrine system* (exclude neoplasm)
Growth delay or failure to thrive	Growth delay*, Failure to thrive*, Intrauterine growth retardation, Growth abnormality, Short stature
Heart	Cardiomyopathy*, Cardiomegaly, Left ventricular hypertrophy<br>(cardiomegaly and LVH accepted for young patients with no hypertension or other cause)
Kidney	Renal tubular dysfunction*
Vision	Optic atrophy, Retinal dystrophy*, Cataract*<br>(cataract counted in young patients, or if specified as congenital, juvenile, developmental)
Hearing	Sensorineural hearing impairment*

II. Metabolic/Imaging studies (max 4 points)

Elevated lactate (2 points)	Lactic acidosis*, Increased serum lactate
Elevated lactate/pyruvate ratio	Elevated lactate:pyruvate ratio
Elevated alanine (2 points)	Abnormal circulating alanine concentration, hyperalaninemia
Elevated CSF lactate (2 points)	Abnormal CSF lactate level, Increased CSF lactate
Elevated CSF protein	Increased CSF protein
Elevated CSF alanine (2 points)	Abnormal CSF alanine concentration, Increased CSF alanine concentration
Urinary tricarboxylic acid excretion (2 points)	*Not in HPO – can score 1 for abnormality of Krebs cycle metabolism*
Krebs cycle intermediates	Abnormality of Krebs cycle metabolism*
Stroke-like picture/MRI	Stroke-like episodes
Leigh syndrome/MRI (2 points)	Abnormal basal ganglia MRI signal intensity, Bilateral basal ganglia lesions, Basal ganglia necrosis, Basal ganglia calcification
1 point added for less specific MRI findings	Abnormal brainstem MRI signal intensity, Focal white matter lesions, Leukoencephalopathy, Abnormality of cerebral white matter, Abnormality of brainstem white matter, Abnormality of globus pallidus, Abnormality of midbrain morphology, Abnormality of caudate nucleus morphology
Elevated lactate/MRS	Abnormal brain lactate level by MRS, elevated brain lactate level by MRS

III. Morphology (max 4 points)

Ragged red/blue fibers (score 4)	Ragged-red muscle fibers
COX-negative fibres (score 4)	Cytochrome C oxidase-negative muscle fibers
Reduced COX staining (score 4)	*Not in HPO*
Reduced SDH staining	*Not in HPO*

SDH positive blood vessels (score 2)	*Not in HPO*
Abnormal mitochondria on EM (score 2)	Abnormal mitochondrial morphology*
Abnormal respiratory chain enzymology	Decreased activity of mitochondrial respiratory chain*
Depletion of mtDNA, Multiple deletions of mtDNA (in patient <60 years for both)	Depletion of mitochondrial DNA in muscle, Depletion of mitochondrial DNA in liver, Multiple mitochondrial DNA deletions (in patient <60 years)

<u>Notes</u>

- The list is not exhaustive but based on frequently used HPO terms within the cohort of suspected mitochondrial disorder patients in the 100 000 Genomes Project.
- The HPO term 'stroke-like episodes' was counted as a clinical sign/symptom AND as an MRI finding.
- A list of HPO terms relating to MRI findings which are suggestive of a mitochondrial disorder but less specific are scored as 1 point.
- Respiratory chain enzymology was added to the modified Nijmegen criteria as used by Riley et al[7]. Due to using HPO terms, we have scored this as 1 point, rather than the 1 or 2 points depending on the level of activity.
- We added 1 point for depletion of mtDNA or multiple mtDNA deletions in patients under 60 years.

Supplementary Table D – Gene Panels Applied

Panel Name	Number of participants
Mitochondrial disorders	345
Undiagnosed metabolic disorders	148
Intellectual disability	139
Congenital myopathy	77
Hereditary ataxia	60
Early onset dystonia	59
Distal myopathies	59
Epileptic encephalopathy	51
Congenital hearing impairment (profound/severe)	45
Hereditary spastic paraplegia	44
Brain channelopathy	40
Structural basal ganglia disorders	34
Inherited white matter disorders	33
Genetic epilepsy syndromes	33
Hearing loss	28
Congenital disorders of glycosylation	26
Hereditary neuropathy	24
Congenital muscular dystrophy	24
Skeletal muscle channelopathies	22
Rare multisystem ciliopathy disorders	22
Charcot-Marie-Tooth disease	21
Cerebellar hypoplasia	20
Optic neuropathy	17
Inherited optic neuropathies	15
Cataracts	15
Rhabdomyolysis and metabolic muscle disorders	12
RASopathies	12
Paediatric motor neuronopathies	12
Limb girdle muscular dystrophy	11
Congenital myaesthenic syndrome	11
Congenital myaesthenia	10
Rental tubular acidosis	9
Parkinson disease and complex parkinsonism	8
Hypertrophic cardiomyopathy	8
Familial hypercholesterolaemia	8

Hypertrophic cardiomyopathy – teen and adult	7
Unexplained skeletal dysplasia	6
Retinal disorders	6
Intracerebral calcification disorders	6
Dilated cardiomyopathy and conduction defects	6
Skeletal dysplasia	5
Left ventricular noncompaction cardiomyopathy	5
Posterior segment abnormalities	4
Neonatal and familial gastrointestinal neuromuscular disorders	4
Gastrointestinal neuromuscular disorders	4
Dilated cardiomyopathy – teen and adult	4
Peroxisomal disorders	3
Malformations of cortical development	3
IUGR and IGF abnormalities	3
Dilated cardiomyopathy	3
Severe microcephaly	2
Primary microcephaly – microcephalic dwarfism spectrum	2
Extreme early-onset hypertension	2
Diabetes with additional phenotypes suggestive of a monogenic aetiology	2
Cystic kidney disease	2
Clefting	2
CAKUT	2
Autosomal recessive congenital ichthyosis	2
Arthrogryposis	2
Arrythmogenic right ventricular cardiomyopathy	2
Amyotrophic lateral sclerosis/ motor neuron disease	2
Vici syndrome and other autophagy disorders	1
Severe early-onset obesity	1
Renal tract calcification (or nephrolithasis/ nephrocalcinosis)	1
Proteinuric renal disease	1
Primary ovarian insufficiency	1
Primary immunodeficiency	1
Periodic fever syndromes	1
Palmoplantar keratoderma and erythrokeratodermas	1
Insulin resistance (including lipodystrophy)	1
Neurotransmitter disorders	1
Neonatal cholestasis	1
Mucopolysaccharidosis, Gaucher, Fabry	1
Monogenic nephrogenic diabetes insipidus	1

Ketotic hypoglycaemia	1
Inherited bleeding disorders	1
Infantile enterocolitis & monogenic inflammatory bowel disease	1
Hypogonadotrophic hypogonadism	1
Hyperammonaemia	1
Glaucoma (developmental)	1
Fetal hydrops	1
Familial non syndromic congenital heart disease	1
Familial genetic generalised epilepsies	1
Familial diabetes	1
Familial cerebral small vessel disease	1
Early onset dementia (encompassing fronto-temporal dementia and prion disease)	1
Disorders of sex development	1
Corneal abnormalities	1
Cerebral folate deficiency	1
Beckwith-Wiedemann Syndrome (BWS) and other congenital overgrowth syndromes	1
Anophthalmia or microphthalmia	1
Adult solid tumours for rare disease	1

Supplementary Table E – Genomic coordinates for short tandem repeat regions for the 13 short tandem repeat genes analysed

Curated coordinates for json files (EHv3.2.2). Genomic coordinates defined for the region where the repeat motif is located in each gene. Different specifications have been used for GRCh37 and GRCh38 human genome assemblies when running ExpansionHunter v3.1.2. Coordinates corresponding to *AR*, *ATN1*, and *ATXN3* have been updated from the GitHub repository (https://github.com/Illumina/ExpansionHunter) for this analysis to match the repeats targeted by OCR.

STR gene	Repeat motif	Coordinates GRCh37	Coordinates GRCh38
AR	(GCA)*	X:66765161-66765227	chrX:67545319-67545385
ATN1	(CAG)*	12:7045891-7045936	chr12:6936728-6936773
ATXN1	(TGC)*	6:16327867-16327954	chr6:16327636-16327723
ATXN2	(GCT)*	12:112036753-112036822	chr12:111598949-111599018
ATXN3	(GCT)*	14:92537344-92537386	chr14:92071000-92071042
ATXN7	(GCA)*(GCC)+	3:63898360-63898390<br>3:63898390-63898402	chr3:63912684-63912714<br>chr3:63912714-63912726
C9orf72	(GGCCCC)*	9:27573526-27573544 + off target regions	chr9:27573528-27573546 + off target regions
CACNA1A	(CTG)*	19:13318672-13318711	chr19:13207858-13207897
DMPK	(CAG)*	19:46273462-46273522	chr19:45770204-45770264
FMR1	(CGG)*	chr9:27573528-27573546 + off target regions	chrX:147912050-147912110 + off target regions
FXN	(A)*(GAA)*	9:71652177-71652202<br>9:71652202-71652220	chr9:69037261-69037286<br>chr9:69037286-69037304
HTT	(CAG)*CAACAG(CCG)*	4:3076603-3076660<br>4:3076666-3076693	chr4:3074876-3074933<br>chr4:3074939-3074966
PPP2R2B	(GCT)*	5:146258290-146258320	chr5:146878727-146878757
TBP	(GCA)*	6:170870991-170871105	chr6:170561906-170562017

Supplementary Table F – Repeat-size threshold for premutation for the 13 short tandem repeat genes analysed

Locus	Threshold (repeat count)
AR	35
ATN1	35
ATXN1	38
ATXN2	30
ATXN3	44
CACNA1A	17
ATXN7	17
C9orf72	30
DMPK	50
FMR1	55
FXN	44
HTT	35
TBP	40

Supplementary Table H - Potentially Treatable Disorders identified in this study

**Gene**	**Disorder**	**Potential Treatment**
**Mitochondrial Disorders**		
AIFM1	Combined oxidative phosphorylation deficiency type 6	Riboflavin supplementation
HIBCH	HIBCH-deficiency	Low-valine diet
PDHA1	Pyruvate dehydrogenase E1-alpha deficiency	Thiamine and ketogenic diet
PDP1	Pyruvate dehydrogenase phosphatase deficiency	Thiamine and ketogenic diet
**Non-mitochondrial Disorders**		
AMACR	Alpha-methylacyl-CoA racemase deficiency	Dietary exclusion of pristanic and phytanic acid
CACNA1A	Developmental and epileptic encephalopathy (type 42)	Trial of acetazolamide or verapamil
GCDH	Glutaricaciduria, Type 1	Low lysine diet, carnitine supplementation
HADHA	LCHAD deficiency	Low long chain fat diet, medium chain triglyceride supplementation, low dose carnitine
MYO9A	Congenital myasthenic syndrome	Pyridostigmine
SLC52A2	Riboflavin transporter deficiency	High dose riboflavin
TTR	Hereditary Transthyretin-related amyloidosis	Referred to National Amyloidosis Centre for specialised treatment

Supplementary Table I – Sensitivity and specificity of HPO-modified Mitochondrial Disease Criteria Score to differentiate between mitochondrial and non-mitochondrial disorders

Sensitivity= True Positive/ (True Positive + False Negative)

Specificity= True Negative/(True Negative + False Positive)

I. Using MDC score >=5 (probable and definite categories)

	Confirmed genetic diagnosis of mitochondrial disorder (nuclear or mtDNA)	Confirmed genetic diagnosis of a non-mitochondrial monogenic disorder
MDC score >=5	True positive<br>22	False Positive<br>19
MDC score <=4	False Negative<br>8	True Negative<br>31

Sensitivity = 22/30 (73%)

Specificity = 31/50 (62%)

II. Using MDC score >=8 (definite category)

	Confirmed genetic diagnosis of mitochondrial disorder (nuclear or mtDNA)	Confirmed genetic diagnosis of a non-mitochondrial monogenic disorder
MDC score >=8	True Positive<br>12	False Positive<br>3*
MDC score <=7	False Negative<br>18	True Negative<br>47

Sensitivity = 12/30 (40%)

Specificity = 47/50 (94%)

*These participants had variants in *SCN2A* (family 89), *CACNA1A* (family 55) and *TANGO2* (family 95).

Supplementary References

1. Alston CL, Veling MT, Heidler J, Taylor LS, Alaimo JT, Sung AY, et al. Pathogenic Bi-allelic Mutations in NDUFAF8 Cause Leigh Syndrome with an Isolated Complex I Deficiency. Am J Hum Genet. 2020 02;106(1):92–101.

2. Bugiardini E, Mitchell AL, Rosa ID, Horning-Do H-T, Pitmann AM, Poole OV, et al. MRPS25 mutations impair mitochondrial translation and cause encephalomyopathy. Hum Mol Genet. 2019 15;28(16):2711–9.

3. Rahikkala E, Myllykoski M, Hinttala R, Vieira P, Nayebzadeh N, Weiss S, et al. Biallelic loss-of-function P4HTM gene variants cause hypotonia, hypoventilation, intellectual disability, dysautonomia, epilepsy, and eye abnormalities (HIDEA syndrome). Genet Med. 2019;21(10):2355–63.

4. Okur V, Cho MT, van Wijk R, van Oirschot B, Picker J, Coury SA, et al. De novo variants in HK1 associated with neurodevelopmental abnormalities and visual impairment. Eur J Hum Genet EJHG. 2019;27(7):1081–9.

5. Morava E, van den Heuvel L, Hol F, de Vries MC, Hogeveen M, Rodenburg RJ, et al. Mitochondrial disease criteria: diagnostic applications in children. Neurology. 2006 Nov 28;67(10):1823–6.

6. Witters P, Saada A, Honzik T, Tesarova M, Kleinle S, Horvath R, et al. Revisiting mitochondrial diagnostic criteria in the new era of genomics. Genet Med Off J Am Coll Med Genet. 2018;20(4):444–51.

7. Riley LG, Cowley MJ, Gayevskiy V, Minoche AE, Puttick C, Thorburn DR, et al. The diagnostic utility of genome sequencing in a pediatric cohort with suspected mitochondrial disease. Genet Med Off J Am Coll Med Genet. 2020 Apr 21;

8. Robinson PN, Köhler S, Bauer S, Seelow D, Horn D, Mundlos S. The Human Phenotype Ontology: a tool for annotating and analyzing human hereditary disease. Am J Hum Genet. 2008 Nov;83(5):610–5.

9. Köhler S, Doelken SC, Mungall CJ, Bauer S, Firth HV, Bailleul-Forestier I, et al. The Human Phenotype Ontology project: linking molecular biology and disease through phenotype data. Nucleic Acids Res. 2014 Jan;42(Database issue):D966-974.

10. WebHome < MITOMAP < Foswiki [Internet]. [cited 2020 Dec 16]. Available from: https://www.mitomap.org/MITOMAP

11. Wei W, Gomez-Duran A, Hudson G, Chinnery PF. Background sequence characteristics influence the occurrence and severity of disease-causing mtDNA mutations. PLoS Genet. 2017;13(12):e1007126.

12. Yarham JW, Al-Dosary M, Blakely EL, Alston CL, Taylor RW, Elson JL, et al. A comparative analysis approach to determining the pathogenicity of mitochondrial tRNA mutations. Hum Mutat. 2011 Nov;32(11):1319–25.
